# Interplay Between Helminth Infections, Malnutrition, and Gut Microbiota in Children and Mothers from Pemba, Tanzania: Potential of Microbiota-Directed Interventions

**DOI:** 10.3390/nu16234023

**Published:** 2024-11-24

**Authors:** Aristide Toussaint Nguélé, Matteo Mozzicafreddo, Chiara Carrara, Angela Piersanti, Salum Seif Salum, Said M. Ali, Cristina Miceli

**Affiliations:** 1 School of Biosciences and Veterinary Medicine, University of Camerino, 62032 Camerino, Italy; aristidetoussaint.nguele@unicam.it (A.T.N.); chiara.carrara@studenti.unicam.it (C.C.); angela.piersanti@unicam.it (A.P.); 2 Institut Supérieur des Sciences de la Santé, Université Adventiste Cosendai, Nanga Eboko 04, Cameroon; 3 Department of Clinical and Molecular Sciences, Marche Polytechnic University, 60126 Ancona, Italy; m.mozzicafreddo@staff.univpm.it; 4 Department of Biology, University of Padova, 35121 Padova, Italy; 5 School of Health and Medical Sciences, State University of Zanzibar, Zanzibar City 146, Tanzania; salummchenga@yahoo.com; 6 Public Health Laboratory Ivo de Carneri, Chake Chake 122, Tanzania; saidmali2003@yahoo.com

**Keywords:** malnutrition, stunting, wasting, underweight, helminth, *Ascaris*, *Trichuris trichiura*, gut microbiota, children, women of reproductive age

## Abstract

Background/Objectives: Despite efforts within the framework of the Sustainable Development Goal to end malnutrition by 2030, malnutrition and soil-transmitted helminth infections persist in sub-Saharan Africa. A significant barrier to success is the inadequate understanding of effective intervention methods. Most research on the gut microbiota’s role in health has been conducted in developed countries, leaving a critical gap in knowledge regarding low-income populations. This study addresses this gap by expanding research on the gut microbiota of underprivileged populations to help tackle these public health challenges. Methods: We employed 16S rDNA sequencing to assess the bacterial gut microbiota composition of 60 children (mean age: 26.63 ± 6.36 months) and their 58 mothers (mean age: 30.03 ± 6.31 years) in Pemba, with a focus on helminth infection and nutritional status. Results: Our differential abundance analysis identified bacterial taxa that were significantly negatively associated with both helminth infections and malnutrition, highlighting the potential for microbiota-directed interventions to address these health issues simultaneously. Notably, we identified *Akkermansia*, *Blautia*, *Dorea*, and *Odoribacter* as promising probiotic candidates for such interventions. In stunted children, positive co-occurrences were observed between *Lactobacillus*, *Prevotella*, and *Bacteroides*, while *Escherichia/Shigella* displayed negative co-abundance relationships with short-chain fatty acid (SCFA) producers in the gut microbiota. These findings suggest that administering *Lactobacillus* and SCFA-producing probiotics to children may foster the growth of beneficial bacteria like *Prevotella* and *Bacteroides* while reducing the relative abundance of *Escherichia/Shigella*, potentially enhancing overall health. Conclusions: This study underscores the importance of microbiota-directed interventions in children and women of reproductive age as promising strategies, alongside established approaches, for combating helminth infections and malnutrition in vulnerable populations.

## 1. Introduction

Malnutrition remains one of the important world health challenges pointing to the immense challenge of achieving the Sustainable Development Goal (SDG) that aims to eliminate hunger by 2030. Millions of children under five years of age continue to suffer from stunting, wasting, and underweight [1]. Along with the increased proportion of undernourished people, there is also the increasing prevalence of overnutrition, leading to obesity and related health problems. This is called the double burden of malnutrition [2].

Growth faltering in children in the form of stunting, a sign of chronic malnutrition, and wasting, an indicator of acute malnutrition, are common among young children in low- and middle-income countries (LMICs) and may contribute to child mortality and adult morbidity. Three out of five subregions with high rates (more than 30%) of child stunting are found in sub-Saharan Africa: western Africa, middle Africa and eastern Africa [2].

In 2018, in the specific case of Tanzania, the level of stunting was considered “very high” (≥30%) in 15 regions out of 26. In Zanzibar, the 2018 survey indicated prevalences of 21.5%, 5.3%, and 13.6% for child stunting, wasting, and underweight, respectively (*Tanzania National Nutrition Survey (TNNS) 2018*) [3].

Another public health challenge that rages in developing countries, particularly in sub-Saharan Africa, is the soil transmitted helminth (STH) infection. STH infections impact human health, nutrition, and worker productivity, and hence aggravate poverty [4]. Parasitic infections have been recognized for decades to be major public health problems in Zanzibar [5]. Many programs aimed at combating parasitic infections have been implemented in the area, yielding encouraging results in reducing the intensity and morbidity associated with helminth infections. However, the ultimate goal of eliminating STH infections has yet to be achieved [6].

The above-mentioned public health problems are interconnected with synergistic consequence [7]. Helminth infections have been reported to play an important role in malnutrition by causing protein-energy malnutrition, anemia, and physical complications as a result of increased nutrient squandering, blood loss, intestinal obstruction, and rectal prolapse [8]. Many studies have reported that STH infections are significantly associated with undernutrition [9,10,11]. On the other hand, being malnourished is considered as a risk factor for intestinal parasite infection [12]. Another important point to emphasize is that malnutrition has an intergeneration impact [13]. Research suggests that the impact of stunting on development can extend into the next generation of children [14]. Thus, early intervention, particularly in young children and women of childbearing age, is crucial to breaking this cycle [15].

The modest effects of interventions to prevent malnutrition may be attributed to an incomplete understanding regarding the most effective strategies and timing for their implementation [16]. With the development of high throughput sequencing tools and techniques, the study of the gut microbiota and its connection to health and disease has become widely adopted. Therefore, the gut microbiota is now recognized as playing an important role in nutritional conditions [17,18]. Studies have revealed that malnourished children have distinct microbiome compared to healthy counterparts [19,20]. The gut microbiota of children who are undernourished is usually immature, resembling that of younger children rather than that of age-matched healthy controls [21]. Laursen et al. demonstrated that inadequate maturation of the gut microbiota is associated with poor growth and development in early life [22]. These findings suggest a causal relationship between the immature gut microbiota from undernourished infants and impaired growth phenotypes. This was demonstrated by the fact that the transfer and invasion of healthy microbiota into the guts of the undernourished mice by co-housing restored normal growth in the latter [23]. Hopefully, some interventional studies targeting the gut microbiota had positives outcomes. An experimental study using the probiotic *Lactobacillus plantarum* revealed that the gut microbiota enhanced sensitivity to growth hormone [24]. Similarly, Michael et al. found that restoring *Bifidobacterium infantis* to the gut of malnourished infants boosts weight gain and reduces inflammatory markers [25]. Furthermore, there have been many demonstrations that helminth infections shape the gut microbiota composition and its function [26,27,28]. Helminth colonization in the gut requires support from the gut microbiota. Helminth egg hatching is supported by some gut microorganisms such *Escherichia/Shigella* and *Salmonella typhimuriun* [29,30,31]. On the other hand, the success of chemotherapy against helminth infection may be dependent on the composition of the gut microbiota prior to treatment [32].

Despite increasing interest in the study of gut microbiota and its associations with health and disease, research in less-developed countries remains limited. Browne et al. [33] reported that around 85% of the 25,000 high-resolution gut metagenomes publicly available from children under four came from individuals living in wealthy regions (Europe and Nord America). Research indicates that the gut microbiota vary by geographical region where individuals live [34,35], highlighting the urgent need to study the microbiota of less favored populations to assess the effectiveness of microbiota-directed interventions involving them.

This study was conducted in a region with a population vulnerable to malnutrition and parasitic infections, with two primary objectives. First, it aimed to investigate the relationship between helminth infection and gut microbiota shortly after deworming treatment, in order to identify potential protective bacteria that could serve as candidate probiotics or targets for microbiota-directed interventions. Second, it sought to examine the association between malnutrition and gut microbiota to uncover potential protective bacteria that could aid in combating malnutrition, particularly in children, by serving as candidate probiotics or intervention targets.

## 2. Materials and Methods

### 2.1. Ethic Statements

This study was authorized by the Zanzibar Health Research Ethical Committee (ZAHREC/03/REC/MARCH/2022/16), and all participants signed the informed consent form for themselves and their children to participate in the study.

### 2.2. Study Design and Recruitment of Participants

This cross-sectional study was conducted in the Zanzibar archipelago (Tanzania) where helminth infection and malnutrition are endemic. A total of 58 women of reproductive age (WRA), already described in our previous article [36], and their 60 children, described here for the first time, were recruited from three health facilities in Pemba. All participants were either healthy or infected by helminths after the microscopical examination of eggs in their stool samples. A comparative analysis of their gut microbiota was conducted considering the helminth infection and nutritional status.

Inclusion criteria were as follows: (i) All participating mothers were between 18 and 45 years old, and children were 1.5 to 3 years old; and (ii) participants had not taken antibiotics or probiotics in the past two months and did not present any symptoms of disease. All participants not meeting the inclusion criteria were not considered in our study.

A detailed questionnaire was filled in by WRA for collecting information about their lifestyle, family, health, and their nutritional conditions. Nutritional-related anthropometric parameters were measured. Participants meeting the inclusion criteria were provided with stool containers to collect stool samples.

### 2.3. Fecal Sample Collection and Parasitological Analysis

After collection, stool samples were sent and processed at the Public Health Laboratory Ivo De Carneri (PHL-IDC) in Pemba for parasitological analysis. Each sample was divided into aliquots and stored at −20 °C before shipment to the University of Camerino, Italy for the DNA extraction. The Mini–FLOTAC technique was used for microscopic examination. Briefly, two grams of stool sample was homogenized sufficiently with the flotation solution (saturated sodium chloride). After homogenization, the samples were added to the two flotation chambers. After 10 min, the numbers of eggs per gram of feces were determined under a microscope. Analytic sensitivity could reach ten eggs per gram of feces. This analysis was conducted in duplicate for each sample by two well-trained laboratory technicians. After the parasitological analysis, which was based on egg detection, the results were delivered to the enrolled participants to ensure that they could receive anti-helminth treatment from the PHL-IDC team or attend the sanitary center to receive it.

### 2.4. Recording and Evaluation of Nutritional Conditions

With the assistance of nurses, anthropometric parameters such as height, weight, and abdominal circumference were collected for women and their children. We used the World Health Organization (WHO) indicators to evaluate the nutritional conditions of children (WHO. 2024. malnutrition-in-children) [37]. For children, these indicators were defined as follows:

- *Stunting*—height-for-age < −2 SD of the WHO *Child growth standards* median;
- *Wasting*—weight-for-height < −2 SD of the WHO *Child growth standards* median;
- *Overweight*—weight-for-height > +2 SD of the WHO *Child growth standards* median;
- *Underweight*—weight-for-age < −2 standard deviations (SD) of the WHO *Child growth standards* median.

For women, the nutritional conditions were evaluated based on their BMI values (WHO. 2024. Malnutrition in women) [38] as follows:

- BMI < 18.5: underweight; BMI 18.5–24.9: normal weight; BMI ≥ 25.0: overweight; BMI ≥ 30.0: obesity.

### 2.5. DNA Extraction, PCR, and Sequencing

DNA was extracted using the QIAamp Fast DNA Stool Mini Kit by QIAGEN (Hilden, Germany). The DNA concentration and absorbance of each sample was evaluated by using a NanoDrop™ One/One C Microvolume UV–Vis spectrophotometer from Thermo Fisher Scientific (Waltham, Massachusetts, MA, USA).

Before sending the samples for sequencing, all of the extracted DNA was amplified through conventional PCR. The primers used for the purpose were:

Pro 341F: 5′-TCGTCGGCAGCGTCAGATGTGTATAAGAGACAGCCTACGGGNBGCASCAG-3′

Pro 805R: 5′-GTCTCGTGGGCTCGGAGATGTGTATAAGAGACAGGACTACNVGGGTATCTAATCC-3′

The PCR amplified product was run by gel electrophoresis at 120–124 volts for 24 min, then examined under UV light. A total of 50 ng of purified DNA of each sample was subsequently prepared and sent to BMR Genomics (Padova, Italy) for 16S sequencing. Libraries were generated using the NEBNext^®^ Ultra™ DNA Library Prep Kit (New England Biolabs, Ipswich, MA, USA) following the manufacturer’s recommendations. Library quality was assessed and sequenced on an Illumina MiSeq PE300 platform (Illumina, San Diego, CA, USA).

### 2.6. Bioinformatics and Data Analysis

The QIIME2 (Quantitative Insights into Microbial Ecology, version 2023.5) software was used to analyze the 16S rDNA gene sequences generated from NGS technologies. Briefly, after filtering the low-quality reads (minimum quality score of 25, minimum/maximum length of 200–250, no ambiguous bases allowed, no mismatches allowed in the primer sequence, and no phiX reads/chimeric sequences), all of the remaining sequences were subsequently clustered into operational taxonomic units (OTUs) based on their similarity (>97%) following the DADA2 pipeline included in the QIIME 2 Plugin ‘dada2’ (version 2023.5.0). Samples were evaluated for alpha diversity (microbial diversity within samples) and beta diversity analysis (community diversity divergence between samples). We assessed the statistical significance of alpha and beta diversity metrics by the two-sample *t*-test and Kruskal–Wallis and by the PERMANOVA test, respectively, as implemented in QIIME2. Taxonomic analysis was performed by matching OTU sequences with both the Silva and Greengenes databases. The raw reads were deposited into the NCBI Sequence Read Archive database (SRA accession number: SRP495566, BioProject accession number: PRJNA1088637).

### 2.7. Statistical Analysis

Differential analysis of taxonomy between groups was performed using Welch’s *t*-test in STAMP software (version 2.1.3). *p*-values ≤ 5 × 10^−2^ were considered as significant. A student *t*-test was also used with *p* ≤ 5 × 10^−2^ considered as significant.

To detect bacterial co-occurrences in the gut microbiota of stunted and normally growing children and to construct a microbial network from the correlation matrix, Spearman pairwise correlation coefficient analysis was performed between the OTUs using the *cor* function in R-4.4.2. After converting the results into an adjacency matrix, we compared the absolute values to a threshold of 0.3 to include both positive and negative correlations in the network construction.

## 3. Results

### 3.1. Characteristics of Participants

Details of the participant characteristics are reported in Table 1. A total of 58 WRA and 60 children were examined for their nutritional condition and carriage of helminth parasites. The parasitological analysis, based on egg detection, revealed a prevalence of helminthiasis of 24.13% and 33.33% in the WRA and children, respectively. According to information from the Public Health Laboratory Ivo de Carneri and from the participant interviews, they had been treated by anti-helminthic drugs only one or two months before stool collection. This suggests either a failure of the treatment or a fast reinfestation.

Analyses of the nutritional conditions of participants revealed a prevalence of obesity of 17.24% in mothers and a prevalence of child malnutrition (all indicators taken together) of 56.66% in Pemba.

The investigation examining the relationship between helminth infection and malnutrition in children revealed an increased odds of being stunted (OR = 1.21; 95% CI 0.4 to 3.65) with *Trichuris trichiura* infection. Similarly, there was an elevated risk of underweight status associated with *Trichuris* infection (OR = 2.333; 95% CI 0.70 to 7.75).

### 3.2. Study of the Associations Between Helminth Infection and Bacterial Abundance

The analysis of diversity of the gut microbiota according to helminth infection revealed no significant differences in the alpha and beta diversity of the WRA and children gut microbiota analyzed shortly after deworming (see Figure S1). Since previous analyses [26] on this population, with stool samples collected nearly one year after drug treatment, revealed a modulation of gut microbiota diversity in infected individuals compared to non-infected individuals, the present findings suggest that the administration of the anthelmintic drug may have contributed to this modulation, reducing differences in the gut microbiota between infected and uninfected individuals.

However, the examination of the taxonomic composition differences between infected and non-infected children and WRA, based on the advanced statistical analysis with STAMP (Table 2), revealed that *Akkermansia,* typically associated with a healthy gut microbiota and metabolic activity, *Haemophilus*, and *Alloprevotella* were negatively associated with *Ascaris* infection in both the WRA and children. Additionally, several other genera showed significantly different abundances based on helminth infection status. In children, seven other taxa including two members of the Clostridia class (*Eubacterium ruminatium* and *Flavonifractor*) and the genus *Streptococcus,* which includes the commonly used probiotic *Streptococcus thermophilus*, were more abundant in healthy children compared to the *Ascaris*-infected children. Similarly, in the WRA from Pemba, other taxa (see Table 2) including three from the Clostridia class (*Eubacterium eligens, Lachnospira,* and *UCG-005*), were significantly more abundant in non-infected participants compared to those infected with *Ascaris.* These results suggest the potential significance of the Clostridia class in a microbiota-targeted approach to cope with *Ascaris* infection.

Looking at the association between *Trichuris trichiura* and gut microbiota in WRA and children, we identified notable differences. In children, four taxa belonging to the Clostridia class (*Eubacterium_coprostanoligens*, *Christenellaceae_R_7* group, *Clostridia viadin BB60*_group, and *UCG-002*), along with *Paraprevotella* from the Bacteroidia class, were significantly more abundant in the non-infected group. Conversely, 16 taxa (see Table 2) were more prevalent in the non-infected WRA. Among these, *Odoribacter* was also found to be more abundant in *Ascaris* non-infected children, and the genus *Blautia* was found to be negatively associated with *Trichuris* infection [26] in a study conducted in the same population in 2018. This suggests its potential utility as a candidate probiotic or target for microbiota-directed therapy.

### 3.3. Analysis of the Diversity of the Gut Microbiota According to Nutritional Status

**Figure 1:**
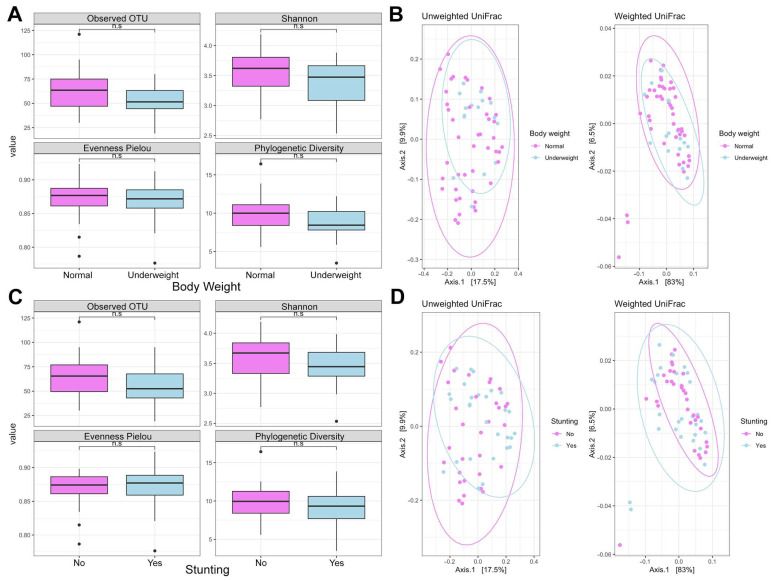
Analysis of the diversity of the gut microbiota of children from Pemba. (**A**) Alpha diversity according to body weight, (**B**) beta diversity according to body weight, (**C**) alpha diversity according to stunting; (**D**) beta diversity according to stunting. “No” refers to “no stunting” while “Yes” indicates “stunted group”.

The diversity analysis uncovered no significant differences in alpha and beta diversity based on child nutritional status. However, alpha diversity was relatively lower in cases of stunting and underweight children, suggesting potential disparities in specific taxa abundances (see Figure 1). Similarly, the analysis did not reveal a significant difference in alpha and beta diversity between the gut microbiota of obese and non-obese WRA (Figure S2).

**Figure 2:**
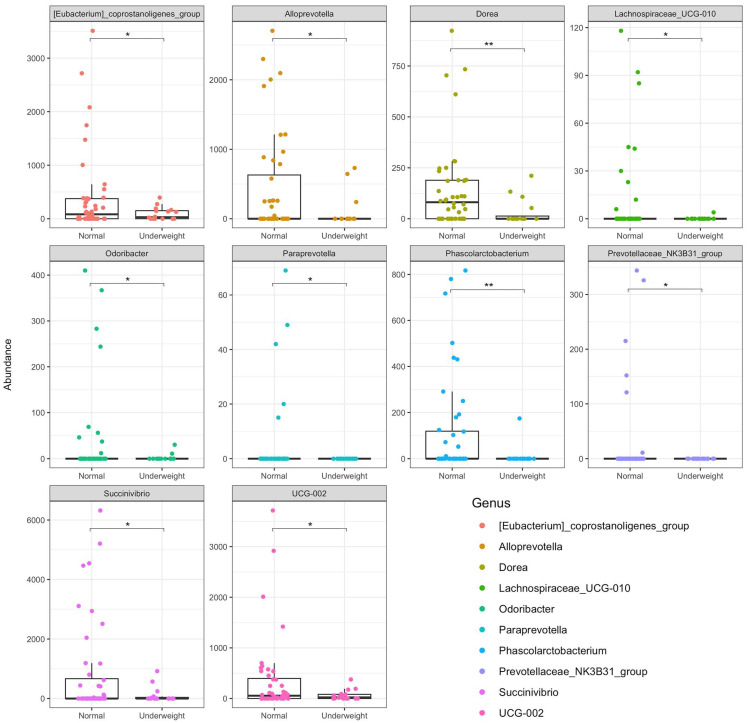
Significant differential abundance of taxa observed by comparing the microbiota of different body weight children from Pemba. * Stands for *p* ≤ 5 × 10^−2^; ** stands for *p* ≤ 1 × 10^−2^.

The analysis of the microbiota based on the children’s body weight (Figure 2) showed that ten genera were significantly more abundant in children with normal weight compared to the underweight group. Most of these taxa belonged to the Bacteroidia class such as *Prevotellaceae_NK3B31*_group (*p* = 3 × 10^−2^), *Alloprevotella* (*p* = 3.9 × 10^−2^), *Paraprevotella*, and *Odoribacter* (*p* = 3.9 × 10^−2^) or to the Clostridia class such as *Dorea* (*p* = 9.23 × 10^−3^), *Eubacterium_coprostanoligenes_group* (*p* = 1.9 × 10^−2^), UCG-002 (*p* = 1.5 × 10^−2^), and *Lachnospiraceae_010* (*p* = 1.9 × 10^−2^). Notably, some of these taxa were also found to be negatively associated with helminth infection in this study: *Alloprevotella* was negatively associated with *Ascaris*; *Eubacterium_coprostanoligenes_group, UCG-002,* and *Paraprevotella* were negatively associated with *Trichuris* infection. Therefore, it can be inferred that a gut microbiota therapeutic approach can be applied to address both helminth infection and malnutrition in children.

**Figure 3:**
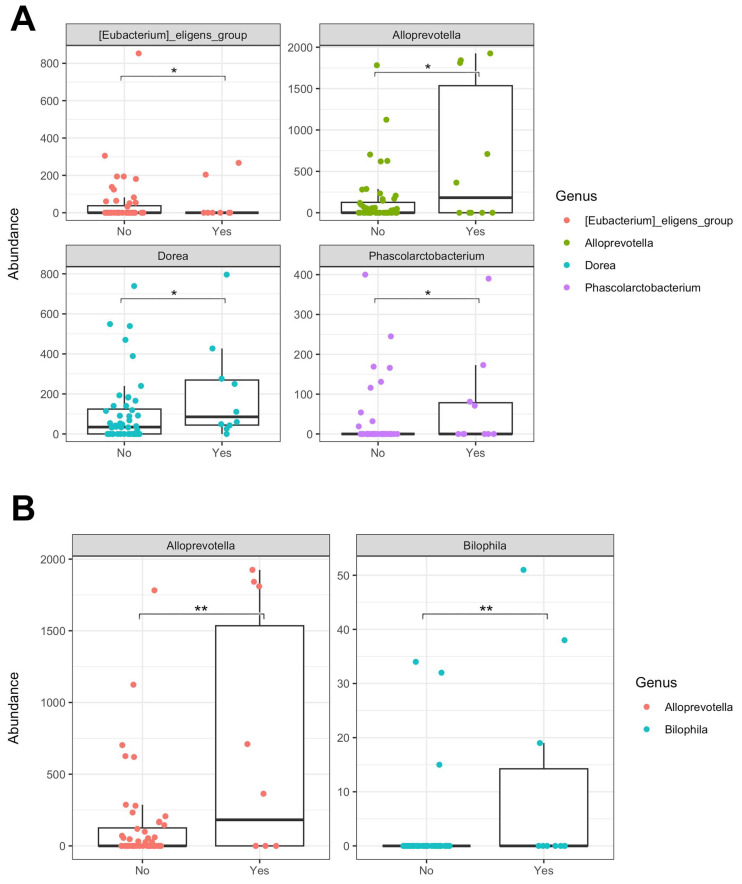
Significant differential abundance of taxa observed by comparing the microbiota of stunted children from Pemba (**A**). “No” refers to “no stunting” while “Yes” indicates the “stunting group”. Significant differential abundance of taxa observed by comparing the microbiota of obese and not-obese women from Pemba (**B**). “No” refers to “not-obese” while “Yes” indicates “obese group”. * Stands for *p* ≤ 5 × 10^−2^; ** stands for *p* ≤ 1 × 10^−2^.

In addition, we conducted a comparative analysis of taxonomy between children with stunting (growth retardation) and those with normal growth. Our analyses identified four taxa that were significantly more abundant in children with normal growth than in the stunted group (see Figure 3A). The genus *Dorea* (*p* = 2.2 × 10^−2^), which was also found to be less abundant in underweight children, was notably less abundant in stunted children, suggesting its potential as a marker of healthy nutritional conditions in children (normal weight and height). *Alloprevotella* (*p* = 4.1 × 10^−2^), less abundant in *Ascaris*-infected children, was also found to be lower in stunted children but significantly more abundant in non-stunted children. Additionally, two other genera, *Phascolarctobacterium* (*p* = 1.2 × 10^−2^) and *Eubacterium_eligens_group* (*p* = 2.1 × 10^−2^), showed similar patterns of abundance.

Furthermore, we analyzed the taxonomy regarding the wasting condition in children (see Figure S3) and found that six taxa were significantly more abundant in the group of children not suffering from wasting compared to children with wasting. The phylum Verrucomicrobiota (*p* = 5 × 10^−3^) was notably higher in the group without wasting, a finding supported by an increased abundance of the genus *Akkermansia*, the preeminent member of the phylum, in the group of children with normal weight. Additionally, four other taxa negatively associated with wasting were identified: *RF39* (*p* = 1.1 × 10^−2^), Christensenellaceae*_R-7_group* (*p* = 1.4 × 10^−2^), *Lachnospira* (*p* = 2 × 10^−2^), and *Haemophilus* (*p* = 1 × 10^−2^).

Finally, we analyzed the significant differences in bacterial abundance regarding the WRA nutritional status (obesity) (Figure 3B). *Alloprevotella* (*p* = 3 × 10^−3^) and *Bilophila* (*p* = 8 × 10^−3^) were significantly more abundant in the obese participants than in the non-obese individuals.

### 3.4. Analysis of Bacteria Co-Occurrences According to the Stunting Condition

**Figure 4:**
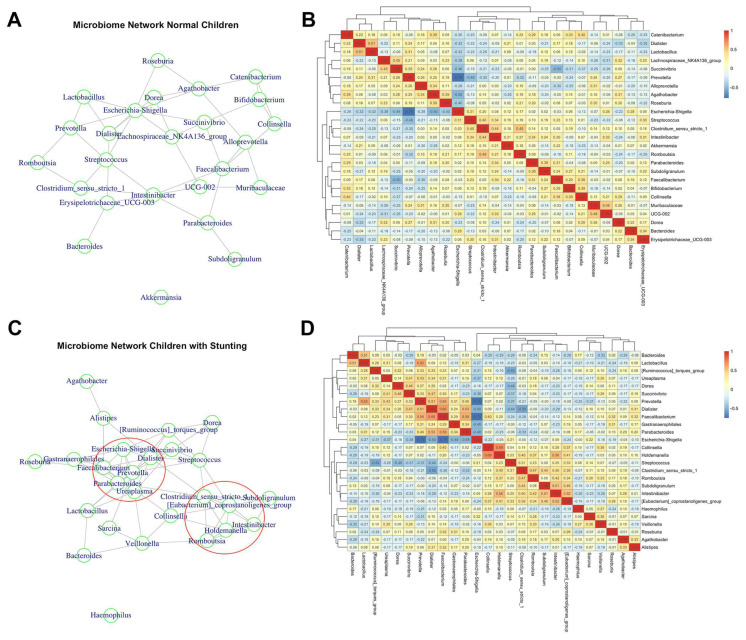
Microbial network and bacterial co-occurrences in normal and stunted children. (**A**) Network in children with normal growth, (**B**) bacteria co-occurrences in normal growth, (**C**) network in stunted children, (**D**) bacteria co-occurrences in stunted children. Potential taxonomic clusters are indicated within the red circle.

To deepen our understanding of stunting’s impact on the children’s microbiota, we analyzed the co-occurrences within these groups, and the results are shown in the microbial networks of both normally growing and stunted children (Figure 4A and Figure 4C, respectively). The data used to construct the networks are shown in the adjacency matrices reported in Figure 4B,D. While the microbial network of normally growing children did not show clear clusters (Figure 4A), thus suggesting a higher sample variability, that of the stunted group (Figure 4C) showed two clusters of coexisting microbes. Relevant positive co-occurrences were observed between *Lactobacillus* and *Dialister* (Figure 4B) and between *Lactobacillus* and *Prevotella* and *Bacteroides* (Figure 4D). This suggests that administering *Lactobacillus* probiotics to children may promote the development of beneficial bacteria in their gut microbiota, potentially enhancing their health. Furthermore, consistent co-occurrences were found in both groups. For instance, positive co-occurrences involved *Clostridium sensu stricto_1*, previously identified as negatively associated with helminth infection, with *Intestinibacter* and *Romboutsia* in both the stunted (lower cluster in Figure 4C,D) and normal groups (Figure 4B). Additionally, *Escherichia/Shigella*, typically linked with helminth and gut bacterial infections, exhibited negative correlations with beneficial and short-chain fatty acid (SCFA) producers, such as *Faecalibacterium*, *Roseburia*, *Prevotella*, and *Agathobacter,* in both normal (Figure 4B) and stunted children (upper cluster in Figure 4C,D). These findings suggest that interventions targeting gut microbiota to reduce *Escherichia/Shigella* proliferation may mitigate its negative impact on healthy bacteria and promote SCFA production, ultimately benefiting the children’s nutritional status and health.

### 3.5. Detection of Enterotypes

**Figure 5:**
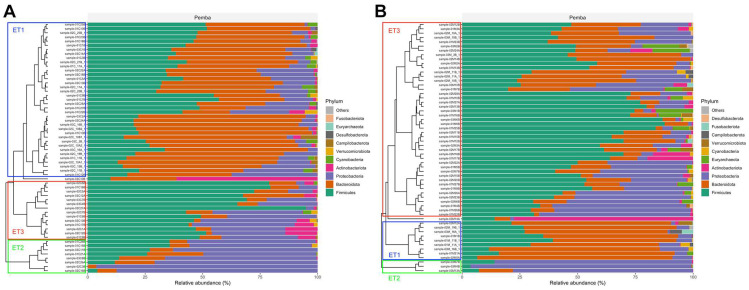
Enterotypes of the gut microbiota. (**A**) Enterotypes in children and (**B**) enterotypes in mothers.

The analysis of the taxonomy revealed that our samples were classified into three distinct clusters, referred to as enterotypes ET1, ET2, and ET3 (Figure 5), according to Schneeberger et al. [32]. ET1 was dominated by Bacteroidetes, ET2 by Proteobacteria, and ET3 by Firmicutes. Children predominantly exhibited enterotype ET1 while mothers showed ET3. Enterotype ET2, dominated by Proteobacteria, was more commonly found in the children than in the mothers. Notably, almost all children in this enterotype group suffered from some form of malnutrition (eight out of nine), with stunting being the most frequently observed condition. No specific association was found between the distribution of helminth infections and the enterotypes. Considering mother–child pairs, only 12 children shared the same enterotype as their mothers: half of them were 18 months old, while the other half were more than 30 months old.

**Figure 6:**
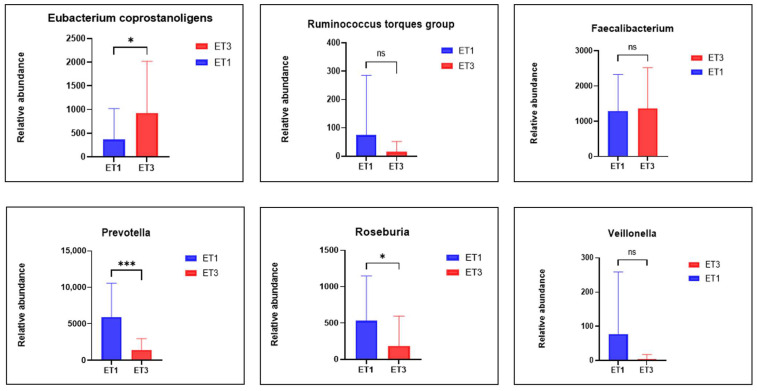
Comparative analysis of selected taxa relative abundances in the enterotypes ET1 and ET3. * stands for *p* ≤ 5 × 10^−2^, *** stands for *p* ≤ 1 × 10^−4^, ns means *p* ≥ 5 × 10^−2^. Means and standard deviations were used in the statistical analysis.

We selected specific taxa previously studied in relation to the response to anthelminthic drugs [32]. We found that the *Eubacterium coprostanoligenes* group, which is part of the enterotype that responds favorably to deworming treatment, was significantly (*p* = 2.3 × 10^−2^) more abundant in enterotype ET3 compared to ET1 (Figure 6). Conversely, *Ruminococcus torques*, also associated with a favorable response to treatment, was relatively less abundant (*p* = 2.21 × 10^−1^) in enterotype ET3. This suggests that microbiota-directed interventions aimed at increasing the abundance of *Ruminococcus torques* should be considered to enhance the microbiota’s responsiveness to treatment in the studied population. On the other hand, *Prevotella* (*p* = 4 × 10^−4^) and *Roseburia* (*p* = 3.3 × 10^−2^) were significantly more abundant in enterotype ET1, with *Veillonella* also relatively more abundant (*p* = 8.5 × 10^−2^) in this group, confirming the enterotype classification shown in [32].

## 4. Discussion

Our findings shed light on the interplay between gut microbiota, helminthiasis, and malnutrition. In the Pemba population, we observed a relatively high percentage of helminth infection despite the recent deworming campaign, alongside a high prevalence of malnutrition among children. Differential abundance analyses uncovered taxa with a negative association with either helminth infection or malnutrition as well as taxa that exhibited negative associations with both helminth infection and malnutrition, suggesting that microbiota-directed interventions could serve as a dual approach to fight these health issues simultaneously.

The relatively high percentage of helminth infection (24.13% and 33.33% in mothers and children, respectively) observed shortly after deworming campaigns suggests either rapid reinfestation or the failure of chemotherapy to effectively eliminate helminth infections in this population. In a cross-sectional study conducted on the same population in 2018, Chen et al. [26] reported similar prevalence rates (37.93% in mothers and 22.22% in children), with samples collected nearly a year after the deworming campaign. These findings closely align with the prevalence observed in our study, which was conducted shortly after a deworming campaign. Previous research on this population has highlighted the limited efficacy of chemotherapy interventions and the reduced sensitivity of *Trichuris trichiura* to commonly used anthelminthic drugs [6,39,40]. This ongoing challenge underscores the urgent need to identify complementary strategies to combat helminth infections in this population.

In addition, this analysis also revealed a high prevalence of malnutrition among children, with an overall rate of 56.66% in Pemba. This finding is comparable to data from Ghana, where nearly half of all school-children were malnourished [8]. These results highlight the severity of child malnutrition in sub-Saharan Africa. The prevalence of child malnutrition in Pemba, marked by stunting (46.66%), underweight (26.66%), and wasting (23.33%) was notably higher than that reported in the 2018 Tanzanian National Nutritional Survey, which documented rates of 21.5%, 5.3%, 13.6% for stunting, wasting, and underweight, respectively. The difference observed here can be attributed to the age range covered in our study, which was 18 to 36 months, compared to the broader range of 0 to 59 months in the national survey. However, it is noteworthy that in the national survey, the prevalence of stunting among children aged 24 to 35 months was similar (43.3%) to the one found in this study (46.66%). This suggests a heightened vulnerability of children aged 1.5 to 3 years—a critical period of nutritional transition, when children gradually shift from maternal feeding to more adult-like feeding habits. This result also alerts that the Global Nutrition Targets by 2025, which aim for a 40% reduction in the number of children aged under 5 years who are stunted [2], may not be achieved without the development of a more appropriate and integrated approach. Moreover, a prevalence of stunting in childhood may result in obesity later on in women’s lives. Henriques et al. demonstrated that being stunted predisposed an individual to obesity [41]. Therefore, fighting against stunting not only save children’s lives, but prevents the later onset of obesity and its related complications.

Our analysis revealed elevated odds of being stunted and underweight for children who had a *Trichuris trichiura* infection. Previous studies have shown that the presence of helminth affects the nutritional status of children [7,12,42,43,44] through different mechanisms such as anorexia [42,45], mucosal damage, vomiting, and diarrhea [46,47]. A study in Brazil revealed that helminthiasis in early childhood was associated with a 4.6 cm reduction in height by the age of seven [48]. Conversely, the use of anthelmintic drugs has been linked to improvements in weight, height, and mild-upper arm circumference [49,50]. These findings indicate the importance of combating helminth infections as a critical step in reducing childhood malnutrition. The analysis of bacteria co-occurrences indicates a strong negative co-abundance between *Escherichia/Shigella* and anti-inflammatory and SCFA producers like *Faecalibacterium* and *Dialister*. This finding aligns with Chen et al. (2020), who observed a similar pattern, where species within the *Escherichia* genus showed positive co-abundance with species with pro-inflammatory properties, and negative co-abundance with species with anti-inflammatory properties like *F. prausnitzii* [51]. Additionally, the positive co-occurrences of *Lactobacillus* with beneficial gut bacteria, such as *Bacteroides* and *Prevotella*, suggest a promising role for *Lactobacillus* probiotics in supporting children’s gut microbiota health. This potential is supported by evidence from studies in both mice [52] and humans [53]. However, it is essential to further investigate these correlations at the species and subspecies levels, as microbial interactions can vary significantly depending on the chosen taxonomic resolution [54].

While previous studies have reported significant changes in alpha and beta diversity of gut microbiota with helminth infection [26,55,56], our study did not find a notable impact on overall biodiversity in children. However, specific taxa showed significant differences between the infected and non-infected participants. Similar to our finding, Gobert et al. [57] observed changes in specific taxa abundance without affecting the overall diversity and noted reduced *Haemophilus* in helminth-infected participants, hinting at its potential protective role. In our analysis, *Akkermansia* and *Alloprevotella* were more abundant in *Ascaris*-non-infected participants, with *Akkermansia muciniphila* previously linked to metabolic benefits and reduced helminth infection [58,59]. Additionally, two members of the Clostridia class (*Eubacterium ruminatium* and *Flavonifractor)* were significantly reduced in children, while three other members of the same class in the mothers *(Eubacterium eligens, Lachnospira,* and *UCG-005)* exhibited a similar reduction in abundance as the *Ascaris*-infected participants. Members of this class were also found to be negatively associated with *Trichuris trichiura.* Other taxa more abundant in children without *Trichuris* belonged to the Clostridia class *(Eubacterium_coprostanoligens, Christenellaceae_R_7 group, Clostridia viadin BB60_group,* and *UCG-002),* suggesting a protective property of this bacteria class. These findings align with previous studies that reported lower proportions of *Clostridia* and *Bacteroidia* in helminth-infected individuals [28,55,60]. These studies suggest that microbiota-directed interventions, such as dietary modifications to enhance these bacterial groups, may offer protective benefits for vulnerable populations. Notably, *Odoribacter*, which was more abundant in the non-infected women, has already shown promise as a probiotic, improving glucose control and inflammation in obese mice [61,62,63]. These taxa could serve as candidates for probiotic development to support gut health in vulnerable populations.

Previous studies have explored the relationship between the gut microbiota and malnutrition and have identified significant differences between cases and controls [17,64]. We observed that some taxa, such as *Alloprevotella*, *Eubacterium_coprostanoligenes*_group, *UCG-002*, and *Paraprevotella*, which are negatively associated with child malnutrition, were also negatively associated with helminth infection. This suggests that intestinal parasites may play an essential role in the chronicity of child malnutrition [65]. Among the taxa that differed according to the nutritional condition in children, *Dorea* attracted our attention, as it appeared to be negatively associated with both underweight and stunting conditions. *Dorea formicigenerans* was associated with weight gain in a microbiota-directed food intervention in children [66]. Two other studies found that species of the same genus were positively correlated to weight and lean mass gain [67,68]. A study by Fontaine et al. [17] revealed that the most discriminative species between healthy and undernourished children were *Bifidobacterium longum* for young children in their first six months while the *Dorea* species, *Faecalibacterium Prausnitzii*, and *Ruminococcus* species were relevant for children from 6 to 24 months. Therefore, bacteria from the genus *Dorea* may be considered as candidate probiotics for addressing childhood malnutrition.

*Akkermansia* was negatively correlated with wasting condition and *Ascaris* infection. A reduced abundance of this genus may prevent maturation of the gut microbiota in children, potentially promoting the wasting condition. Roswell et al. [20] discovered that *Akkermansia*, along with other bacteria usually found in the healthy gut microbiota of adults, increased with age in children. Therefore, in *Ascaris* infection, reducing *Akkermansia* may delay the maturation of children’s gut microbiota. This issue may be solved since it has been demonstrated that the early life consumption of polyphenol [69,70,71] and omega-3 supplementation [72] can promote the development of *Akkermansia.*

Our investigation of the composition of the gut microbiota revealed three main clusters in both the children and mothers, each dominated by a different phylum. Enterotype ET2, dominated by Proteobacteria, was mostly found in the children, especially those with malnutrition. This finding aligns with a study by Nuzha et al. [73] that also found Proteobacteria to dominate the microbiota of malnourished children. Furthermore, only one-fifth of the children shared the same enterotype as their mothers, and these children were either at the early stage of gut microbiota development (18 months old) or nearing the end of this development (31–36 months old). This suggests that the age window between 19 and 30 months may be optimal for probiotic interventions aimed at fostering a healthier adult gut microbiota.

*Eubacterium coprostanoligenes* was identified as one of the most abundant species in enterotype ET3, consistent with the findings of Schneeberger et al. [32], who reported that enterotype ET3 is the most favorable for a positive response to albendazole-ivermectin treatment. In a previous study on the same population [36], we observed positive associations between *Eubacterium coprostanoligenes* and specific foods and micronutrients including cassava, vitamin B6, and folate. This indicates that increasing the intake of these foods and nutrients may enhance the gut microbiota’s responsiveness to deworming treatments.

Our study offers the advantage of being the first to simultaneously investigate the connections between helminth infection, malnutrition, and gut microbiota in young children and mothers in one area where helminthiasis and child malnutrition are prevalent. However, the limited resolution of the 16S rDNA sequencing based on the primers we used, although largely applied in most microbiota-compositional studies, did not allow for the detection for subgenus taxa. Additionally, this study did not consider functional aspects, which could be better addressed in further investigations using metatranscriptomic and metabolomic approaches. Another limitation of this study is the relatively small sample size. As the research was conducted in a specific region of Africa, caution is required when attempting to generalize the findings.

Future studies should involve larger cohorts and interventional designs, where participants follow prescribed diets and supplementation protocols. Such studies should also include functional analyses, utilizing metatranscriptomics and metabolomics to provide deeper insights into the gut microbiota’s role and interactions.

## 5. Conclusions

In this cross-sectional and observational study, we used the 16S rDNA sequencing approach to evaluate the composition of the gut microbiota of children and their mothers from Pemba according to their helminth infection and nutritional status. Our analyses showed that despite efforts made by the local government and partners to combat STH infections and malnutrition, their prevalence remains high. The differential abundance of certain taxa and the analysis of co-occurrences allowed us to postulate that microbiota-directed interventions, through diet and/or probiotics, may aid in combating the public health challenges of childhood malnutrition and helminthiasis. Some taxa negatively associated with either helminth infection (*Akkermansia, Haemophilus*, and *Blautia*) or childhood malnutrition (*Dorea* and *Odoribacter*) have emerged as candidate probiotics. Furthermore, these results outline the importance of promoting a nutritional educational campaign among the population to suggest appropriate and sustainable nutrients to improve their intestinal microbiota and health conditions. Nutritional education campaigns should be organized under the supervision of local authorities prior to the nutritional intervention. This should allow the population to better know how to use locally available foods. This should be part of an integrated approach that should also include the regular use of certified anthelminthic drugs and improvements in water quality, hygiene, and sanitation.

## Figures and Tables

**Table 1:** Characteristics of the participants.

Features	Mothers ^1^	Children
Number of participants	58	60
Mean age ± SD	30.03 ± 6.31 (years)	26.63 ± 6.36 (months)
Mean BMI ± SD (mothers)	24.34 ± 4.88 (kg/m^2^)	/
Helminth infected	14	20
*Ascaris* infected	8	5
*Trichuris* infected	8	18
Co-infected	2	3
Obesity (mothers)	17.24% (10/58)	/
Underweight (mothers)	6.89% (4/58)	/
Overall children malnutrition	/	56.66% (34/60)
Stunting (height/age)	/	46.66% (28/60)
Wasting (weight/height)	/	23.33% (14/60)
Underweight children (weight/age)	/	26.66% (16/60)

^1^ Mothers were the same group of women from Pemba described in [36]. Different parameters are here reported.

**Table 2:** Taxa that were significantly more abundant in the healthy participants compared to the Ascaris- or to Trichuris-infected children and women. The analysis was conducted using the software STAMP, which considers the effect size. *p* ≤ 5 × 10^−2^ was considered as statistically significant and reported as a single value for each taxon.

Healthy vs. *Ascaris* (Children)	*p*. Value	Healthy vs. *Ascaris* (Mothers)	*p*. Value	Healthy vs. *Trichuris* (Children)	*p*. Value	Healthy vs. *Trichuris* (Mothers)	*p*. Value
*Eubacterium ruminatium*	1.5 × 10^−2^	*Eubacterium eligens*	8.87 × 10^−3^	*Eubacterium_coprostanoligens*	4.5 × 10^−2^	*Eubacterium eligens*	8.87 × 10^−3^
*Akkermansia*	1.5 × 10^−2^	*Akkermansia*	2 × 10^−2^	*Christensenellaceae_R_7 group*	3 × 10^−2^	*Eubacterium siraeum*	3.62 × 10^−3^
*Alloprevotella*	3.4 × 10^−2^	*Alloprevotella*	7.87 × 10^−3^	*Clostridia viadin BB60_group*	3.4 × 10^−2^	*Ruminococcus torque*	1.4 × 10^−2^
*Haemophilus*	2 × 10^−2^	*Haemophilus*	5.69 × 10^−3^	*Klebsiella*	4.8 × 10^−2^	*Barneiella*	1.9 × 10^−2^
*Megamonas*	9.05 × 10^−3^	*Lachnospira*	3.7 × 10^−3^	*Paraprevotella*	3.4 × 10^−2^	*Bilophila*	4.8 × 10^−2^
*Barnesiella*	1.2 × 10^−2^	*Odoribacter*	2.9 × 10^−2^	*UCG-002*	3.4 × 10^−2^	*Blautia*	1.3 × 10^−2^
*Clostridia UCG-014*	2.7 × 10^−2^	*Phascolarctobacterium*	3.7 × 10^−2^			*Butirivibrio*	2.4 × 10^−2^
*Erysipelotrichaceae UCG-003*	1.7 × 10^−2^	*Sutterella*	6.44 × 10^−3^			Cyanobacteria	1.58 × 10^−3^
*Flavonifractor*	1.7 × 10^−2^	*UCG-005*	2.4 × 10^−2^			Desulfobacterota	4.2 × 10^−2^
*Streptococcus*	3.5 × 10^−2^	Verrucomicrobiota	2 × 10^−2^			*Eryipelotrichaceae UCG-003*	7.51 × 10^−3^
Verrucomicrobiota	1.2 × 10^−2^					Gastranaerophilale	2.14 × 10^−3^
						*Klebsiella*	2.04 × 10^−3^
						*Lachnospiraceae NK4A136*	4.68 × 10^−3^
						*Odoribacter*	1.5 × 10^−2^
						*Roseburia*	1.5 × 10^−2^
						*Sutterella*	1.7 × 10^−2^
						*UCG-005*	2.5 × 10^−2^

## Data Availability

The original data presented in the study are openly available from the publication of this article in the NCBI Sequence Read Archive database (SRA accession number: SRP495566, BioProject accession number: PRJNA1088637).

## Supplementary Materials

The following supporting information can be downloaded at: https://www.mdpi.com/article/10.3390/nu16234023/s1, Figure S1: Analysis of gut microbiota diversity according to the helminth infection. No significant differences were shown in the alpha and beta diversity of the WRA and children’s gut microbiota analyses performed shortly after deworming. (**A**) Alpha diversity according to the parasite presence in WRA; (**B**) beta diversity according to parasite presence in WRA; (**C**) alpha diversity according to parasite presence in children; (**D**), beta diversity according to parasite presence in children. “No” refers to “no parasite detection” while “Yes” indicates “parasite detection”; Figure S2: Analysis of diversity of the gut microbiota revealed no significant difference in alpha (**A**) and beta (**B**) diversity between the gut microbiota of obese and non-obese WRA. “No” refers to “non-obese” while “Yes” indicates “obese” WRA; Figure S3: Significant differential abundance of taxa observed by comparing the microbiota of wasting children, analyzed at the phylum (**A**) and genus (**B**) levels. “No” refers to “not wasting”, while “Yes” indicates “wasting” condition.
