# Supplementary material for: Interplay Between Helminth Infections, Malnutrition, and Gut Microbiota in Children and Mothers from Pemba, Tanzania: Potential of Microbiota-Directed Interventions
Source: Nutrients. 2024 Nov 24;16(23):4023. doi: 10.3390/nu16234023 (PMC11643142; doi:10.3390/nu16234023)
Supplement: Supplementary file 1 [file nutrients-16-04023-s001.zip › nutrients-3329283-supplementary.pdf]

Supplementary Materials:

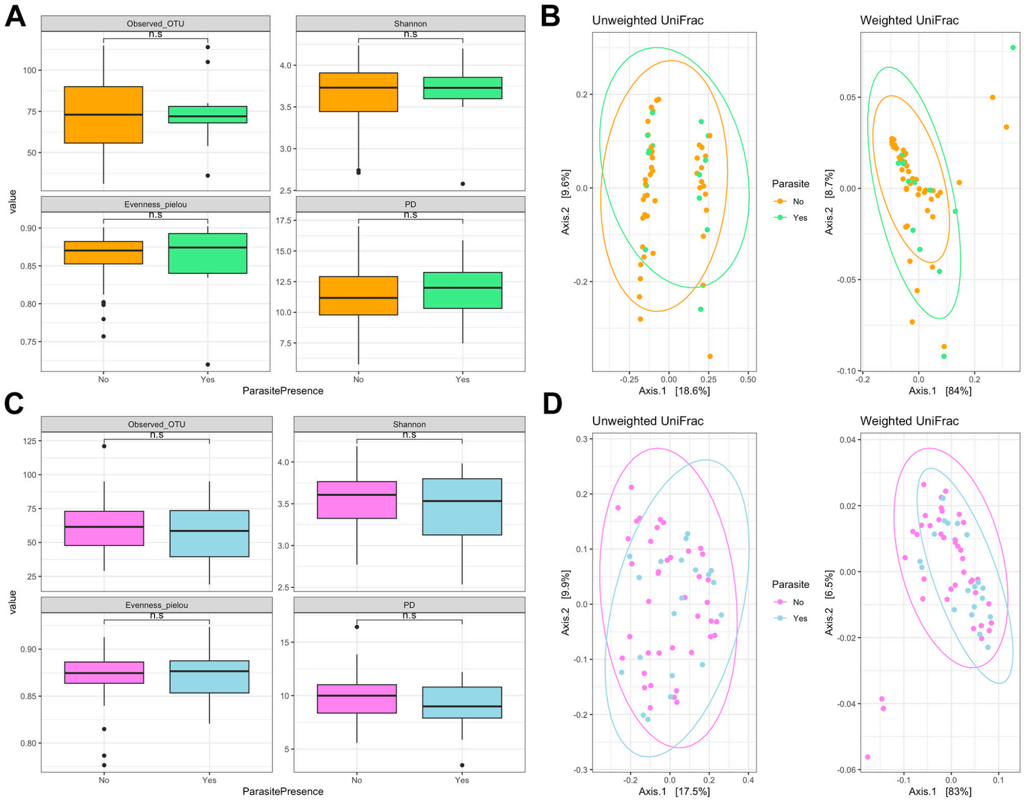

Figure S1. Analysis of gut microbiota diversity according to the helminth infection. No significant differences are shown in the alpha and beta diversity of WRA and children gut microbiota analyses performed shortly after deworming. (**A**), alpha diversity according to the parasite presence in WRA; (**B**), beta diversity according to parasite presence in WRA; (**C**), alpha diversity according to parasite presence in children; (**D**), beta diversity according to parasite presence in children. "No" refers to "no parasite detection" while "Yes" indicates "parasite detection".

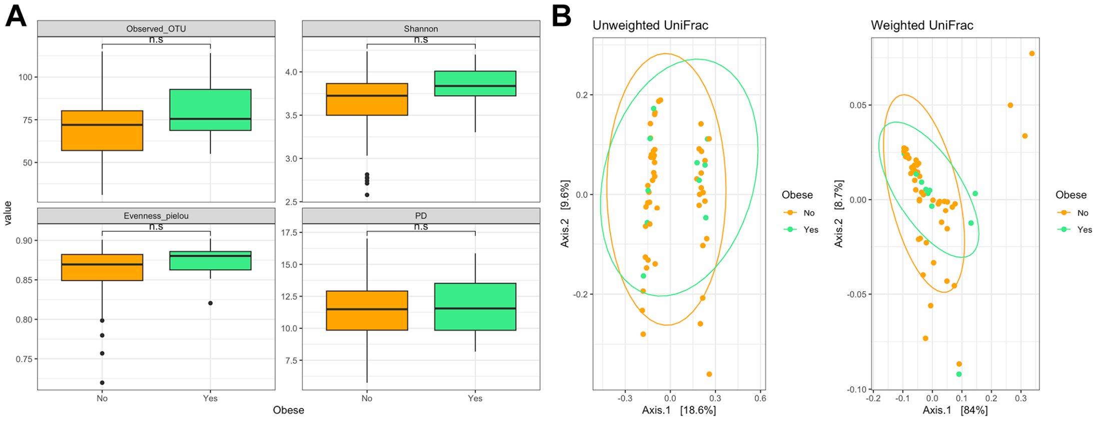

Figure S2. Analysis of diversity of the gut microbiota revealed no significant difference in alpha (**A**) and beta (**B**) diversity between the gut microbiota of obese and non-obese WRA. "No" refers to "non obese" while "Yes" indicates "obese" WRA.

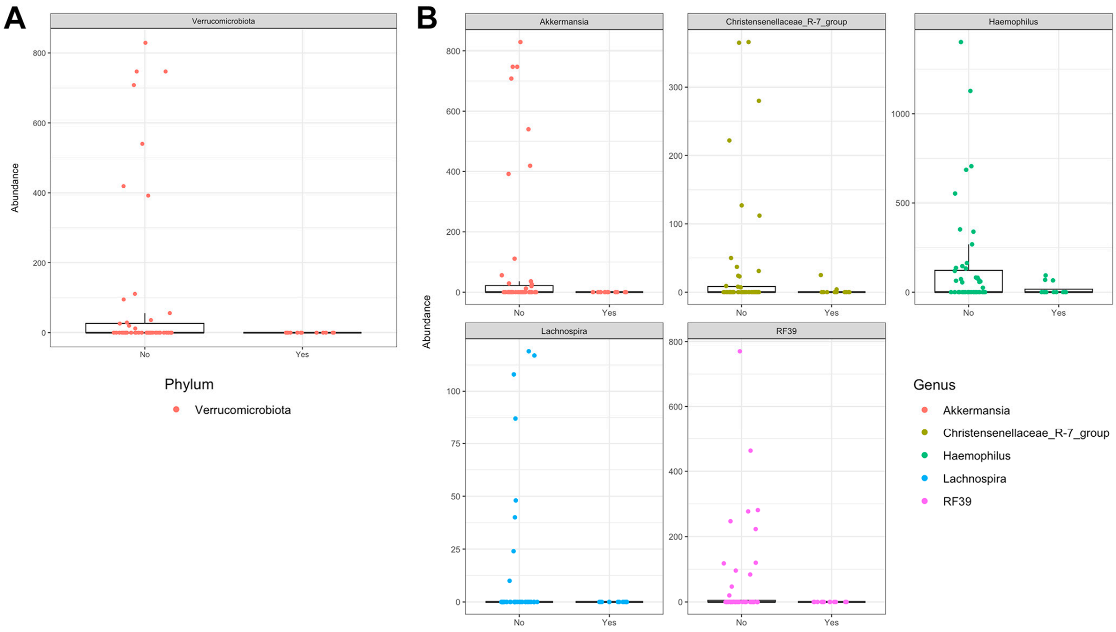

Figure S3. Significant differential abundance of taxa observed by comparing the microbiota of wasting children, analysed at the phylum (**A**) and genus (**B**) levels. “No” refers to “not wasting”, while “Yes” indicates “wasting” condition.
